# Modeling of Magnetoelectric Microresonator Using Numerical Method and Simulated Annealing Algorithm

**DOI:** 10.3390/mi14101878

**Published:** 2023-09-29

**Authors:** Mohammad Sadeghi, Mohammad M. Bazrafkan, Marcus Rutner, Franz Faupel

**Affiliations:** 1 Department of Materials Science, Faculty of Engineering, Kiel University, Kaiserstraße 2, D-24143 Kiel, Germany; mohs@tf.uni-kiel.de; 2 Institute for Metal and Composite Structures, Hamburg University of Technology, Denickestr. 17, D-21073 Hamburg, Germany; mohammad.bazrafkan@tuhh.de (M.M.B.); marcus.rutner@tuhh.de (M.R.)

**Keywords:** magnetoelectric, microresonator, nonlinearity, simulated annealing, numerical simulation, Duffing-oscillator

## Abstract

A comprehensive understanding of the linear/nonlinear dynamic behavior of wireless microresonators is essential for micro-electromechanical systems (MEMS) design optimization. This study investigates the dynamic behaviour of a magnetoelectric (ME) microresonator, using a finite element method (FEM) and machine learning algorithm. First, the linear/nonlinear behaviour of a fabricated thin-film ME microactuator is assessed in both the time domain and frequency spectrum. Next, a data driven system identification (DDSI) procedure and simulated annealing (SA) method are implemented to reconstruct differential equations from measured datasets. The Duffing equation is employed to replicate the dynamic behavior of the ME microactuator. The Duffing coefficients such as mass, stiffness, damping, force amplitude, and excitation frequency are considered as input parameters. Meanwhile, the microactuator displacement is taken as the output parameter, which is measured experimentally via a laser Doppler vibrometer (LDV) device. To determine the optimal range and step size for input parameters, the sensitivity analysis is conducted using Latin hypercube sampling (LHS). The peak index matching (PIM) and correlation coefficient (CC) are considered assessment criteria for the objective function. The data-driven developed models are subsequently employed to reconstruct/predict mode shapes and the vibration amplitude over the time domain. The effect of driving signal nonlinearity and total harmonic distortion (THD) is explored experimentally under resonance and sub-resonance conditions. The vibration measurements reveal that as excitation levels increase, hysteresis variations become more noticeable, which may result in a higher prediction error in the Duffing array model. The verification test indicates that the first bending mode reconstructs reasonably with a prediction accuracy of about 92 percent. This proof-of-concept study demonstrates that the simulated annealing approach is a promising tool for modeling the dynamic behavior of MEMS systems, making it a strong candidate for real-world applications.

## 1. Introduction

Mechanical oscillators are increasingly utilized in metrology systems for monitoring and controlling purposes. Microresonators, intentionally designed for miniaturization, have proven to be indispensable across various fields. Notably, they find applications in frequency filtering within communication systems [1,2], act as sensing elements in atomic force microscopy [3], and serve as accelerometers [4]. Additionally, the unique properties of piezoelectric materials, such as nano-scale resolution, robustness, and fast response rate [5], make them well-suited for precision machining [6,7] and nano-positioning actuators [8,9].

While piezoelectric materials exhibit remarkable capabilities, scholarly interest in magnetostrictive materials has surged, particularly because of their wireless excitation potential in displacement actuators [10]. The combination of magnetostrictive and piezoelectric layers in magnetoelectric composites enables simultaneous magnetic and charge ordering capabilities. This feature unlocks a wide range of applications including energy harvesting [11], mechanical actuators [12], magnetic sensors [13], electric current sensors [14], magnetic particle imaging [15], magnetocardiography [16], and swallowing detection [17].

In the aforementioned applications, the incorporation of closed-loop feedback control to predict system behavior improves the performance of sensors and actuators [18]. Additionally, under certain conditions related to material properties and practical limitations, nonlinear behavior may occur in the system that leads to significant modifications in the operational characteristics. This aspect becomes particularly crucial in the design of micro-electromechanical systems (MEMS), such as microresonators [19]. Microresonators generally function within the linear range, where the vibration amplitude is constrained, resulting in a restricted signal-to-noise ratio. Nonlinearities can have a profound impact on the frequency response of microresonators, influencing their dynamic range and damping behavior in response to changes in the excitation field. As a result, these nonlinear effects can considerably enhance the sensitivity of oscillator devices [20,21]. Vibration energy harvesters and wireless microresonators function at resonance, which leads to amplified output power through the mechanical quality factor. However, the difficulty lies in matching vibration frequencies to the harvester’s resonance frequency, and the sensing range is limited in low bandwidth. As a potential solution, researchers have explored the use of nonlinear mechanical resonators to expand the power bandwidth and sensing range [22,23]. More specifically, for wireless ME antennas [24,25], enhancing data transmission speed involves utilizing a nonlinear signal component to effectively separate information bandwidth and radiation from the antenna’s limited bandwidth. In this regard, the development of a numerical/mathematical model is imperative to gain a comprehensive understanding and to predict the dynamic behavior of microresonators, particularly magnetoelectric actuators.

Recently, researchers have devoted much effort to modeling magnetoelectric MEMS devices using different approaches, such as analytical methods [26,27,28] and numerical simulations [12,13,29]. These endeavors aim to enhance device performance and comprehend nonlinearity behavior [30,31,32,33]. The field-dependent analytical solutions for laminated magnetoelectric composites [26] proposed tunable frequency-multiplying behavior to optimize the piezomagnetic effect. The research findings indicate that when there is no bias field present, only even harmonics are observed. However, in a region with a low-bias field, some odd harmonics become apparent. Furthermore, increasing the bias field to an optimal level enhances the fundamental frequency component in the signal, leading to improved ME sensor linearity. The finite element (FE) analysis of ME heterostructure for ME demonstrates the efficacy of numerical solutions in capturing ME sensor behavior. In this regard, under DC bias conditions, a comparison of the strain, ME coefficient, and voltage was conducted to optimize sensor performance through resonance-enhanced ME coupling. In terms of nonlinearity, micromechanical formulations have been established for the analysis of ME coupling for different composite structures including 1–3, 0–3, and 2–2 connectivities [32]. The study considered the nonlinearity effect when exposed to significant magnetic/electric fields. In micromechanical simulations utilizing the Mori–Tanaka model, it was observed that field-dependent magnetoelectric (ME) responses produced a significant divergence between linear and nonlinear predictions.

The Duffing differential equation could be considered as an underlying model to capture the dynamical behavior of MEMS, which has been reported in several studies [30,33,34,35,36]. Data-driven system identification procedures [36] have emerged as a promising method to reconstruct governing differential equations from recorded vibration signals. In this regard, various machine learning techniques, including sparse identification of nonlinear dynamical systems (SINDy) [35] and artificial neural network (ANN) [37], have been successfully applied to model the systems. However, the application of neural network algorithms in MEMS modeling poses several challenges for real-world scenarios. The issues with excessively large training samples, a high output mean square error, and poor diagnostic precision of backpropagation ANN could be considered in this category [38]. Moreover, the performance of ANN is limited when confronted with slight variations in the system’s input, particularly for vibrations in nonlinear regimes [38]. Despite significant research efforts in the field of MEMS, according to the author’s knowledge, there is still no comprehensive exploration of the dynamic modeling of magnetoelectric microresonators using the data-driven system identification and simulated annealing methods.

The main aim of this study is to investigate the Duffing oscillation of a thin film magnetoelectric microactuator using a simulated annealing algorithm. The vibration behavior of the microresonator is assessed via a laser Doppler vibrometer under various stimulation conditions. Subsequently, an array of mathematical models is reconstructed from the recorded dataset using machine learning method and differential equations. The model’s predictive ability is evaluated through mode shape reconstruction and verification tests. Additionally, the impact of nonlinearity on dynamic behavior is numerically investigated using a finite element simulation. This proof-of-concept study convincingly establishes the effectiveness of the proposed approach in comprehending and manipulating the linearity/nonlinearity behavior of a magnetoelectric microresonator, as evidenced by both numerical simulations and experimental data.

## 2. Material and Methods

### 2.1. Experimental Setup

**Figure 1:**
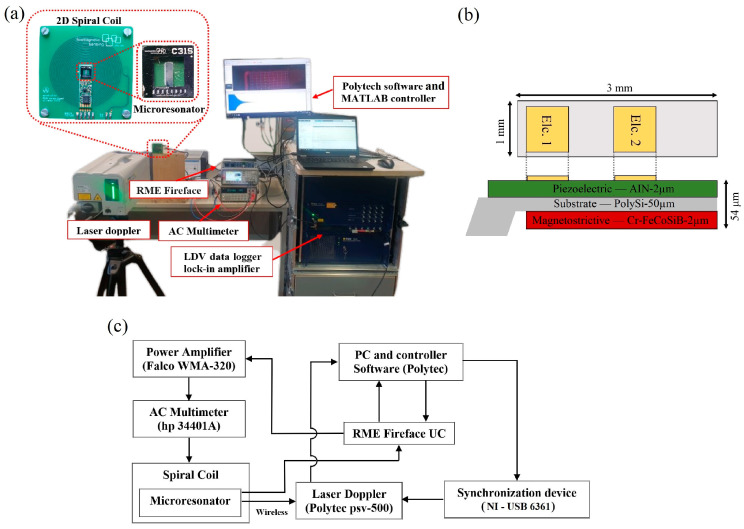
Measurement apparatus; (**a**) fabricated setup, (**b**) schematic structure of devices, (**c**) schematic configuration of microresonator.

The measurement apparatus, including the thin-film microresonator, field generator, displacement recorder, and electric appliances, has been illustrated in Figure 1a. A microresonator, fabricated using wet/dry etching technologies, plays a central role in the measurement setup. This microresonator was made of a thin-film magnetoelectric composite, with a highly magnetostrictive alloy (Cr–FeCoSiB) sputtered in a magnetic field to achieve a thickness of approximately 2 μm. The magnetostrictive layer enabled vibration amplitude manipulation when excited by a wireless magnetic field. In addition, an aluminum nitride (2-μm AIN) layer was sputtered through a low-temperature deposition process, allowing the microresonator to function as a sensor. Additionally, the AIN piezoelectric layer enabled the microresonator to operate under delta-E stimulation mode [39]. The significance of the fabrication procedure for the thin-film actuator lies in the configuration and sequence of deposited layers, both of which exert a notable influence on the output performance, resonance frequency and magnetic noise level of the microresonator [40,41]. To achieve the desired frequency range for the first bending mode with a high-quality factor, a microresonator was designed in the form of a cantilever, measuring 3 mm in length and 1 mm in width. To enhance the stiffness and material compatibility, both magnetostrictive and piezoelectric layers were symmetrically deposited on the 50 μm polysilicon thin film substrate. To utilize the exchange bias effect, the microresonator was subjected to annealing under a strong magnetic field oriented at an optimum angle to the long axis of the cantilever. This approach ensured maximum performance at zero external magnetic bias fields. For further details on the thin-film fabrication procedure, refer to our previous publication [42].

The excitation AC magnetic field was generated using a pair of planar spiral coils. To power the coil, a signal generator (RME Fireface UC, Germany) and an AC power amplifier (WMA-320, Falco system, Netherland) were employed. Continuous measurement of the current was conducted with an AC multimeter (Hp 34401A, Keysight, United States) to guarantee a constant applied driving force. The microresonator was mounted on the top layer of the planar coils. The vibration behavior was recorded using a laser Doppler vibrometer (Psv-500, Polytec, United States). To capture dynamic vibrations on a small surface area, a close-up unit with a micro scan lens (PSV-A-410-x, Polytec, United States) was applied to the LDV device. The laser signal was amplified by an internal lock-in amplifier. The lock-in amplifier is used to separate and extract the particular frequency component of interest and the Doppler-shifted frequency from the signal, which may be influenced by background noise. To ensure synchronization of the excitation signal and laser measurement, a multifunction simultaneous sampling device (NI USB-6361, National Instruments, United States) was used. For a better understanding of the microresonator configuration and electronic appliances used, refer to the schematic diagram shown in Figure 1b,c.

### 2.2. Data-Driven System Identification

Data-driven system identification is the process of reconstructing analytical dynamic models for systems, based on the measured experimental data. It uses statistical and machine learning techniques to describe and ideally predict system behavior without relying on explicit physical equations. This approach proves applicable for complex, nonlinear systems, where deriving precise numerical models is challenging [33,34,35,36].

In this section, the objective function and desired differential equation are described, followed by a comprehensive procedure for system identification and model reconstruction using a machine learning approach.

#### 2.2.1. Duffing Oscillator Operation

The magnetoelectric microresonator operates under an applied wireless driving force that influences the magnetostrictive layer. Due to the nonlinear characterization of the magnetostrictive layer in the ME heterostructure, the displacement vibration is typically modeled using the Duffing equation in a single degree-of-freedom (DOF) system. Previous studies [33,34] have demonstrated the Duffing oscillator’s ability to accurately replicate the dynamic behavior of MEMS oscillation. The general Duffing system under no external force can be modeled as the following non-linear second-order differential equation [43]:

(1) $$\ddot{z}+\zeta \dot{z}+\mu {\dot{z}}^{3}+\alpha z+\gamma z^{3}=0$$

The variable z represents the out-of-plane displacement. Additionally, *ζ* is the damping ratio, *α* is the linear stiffness of the system, *γ* is the non-linear stiffness, and the *μ* coefficient is the non-linear damping term. Under this condition, the system can exhibit three equilibrium points based on the values and signs of the *αγ* parameters. For the purpose of this study, the non-linear damping element is neglected (*μ* = 0), while the driving force is provided by an external magnetic field. The force can be treated as a monofrequency pure signal. Nevertheless, accounting for the noise component and harmonic nonlinearity source in the force signal results in the derivation of the following modified equation:

(2) $$\ddot{z}+\zeta \dot{z}+\alpha z+\gamma z^{3}=F_{r1}cos(\omega t)+F_{r2}cos(2\omega t)+F_{r3}cos(3\omega t)\ldots +n(t)$$

In this context, $F_{r1}$ and ω denote the amplitude and angular frequency of the desired stimulation force, respectively. The contribution of noise, represented by n(t), and the nonlinearity of the excitation signal is controlled by the ratio between $F_{r1}$, $F_{r2}$, and $F_{r3}$ values. Depending on the specific values of the mentioned ratio and the amplitude of $F_{r1}$, the oscillator may either exhibit a chaotic state or a periodic state.

#### 2.2.2. Simulated Annealing Algorithm

Simulated annealing (SA) is a machine learning algorithm first introduced by Kirkpatrick et al. [44]. Inspired by metallurgical annealing processes, SA aims to find the ground state of matter, where the material achieves its minimal energy level. It stochastically explores the solution space and efficiently reaches the minimum condition with limited iterations. SA has demonstrated remarkable success in various fields, including force control [37], manufacturing processes [45], and production optimization [46], showcasing its efficacy for real-world applications.

During each iteration, the algorithm incorporates a minor stochastic alteration to the current solution. The movement to a new point in the solution space is quantitatively assessed by the objective function. Better solutions are directly accepted, while non-improving solutions are accepted based on the cost of the Boltzmann distribution. This approach enables the algorithm to escape local optima and converge toward global optima. The acceptance rate is controlled by the initial temperature and cooling factor parameters, gradually diminishing the acceptance probability of solutions without any enhancements over time. The following equation evaluates the probability of each state based on the objective function [46].

(3) $$P(x)=exp(\frac{-∆f(x)}{kT})$$

where *f*(*x*) is the objective function or system energy, *T* is temperature, and *k* is the Boltzmann constant. More detail about the SA optimization procedure can be found in the literature [47,48,49]. Algorithm 1 demonstrates the SA optimization process that was implemented to reconstruct the Duffing differential equation from the measured dataset. It should be mentioned that the algorithm parameter including initial temperature, cooling rate, and Duffing coefficients’ ranges has been determined via a sensitivity check analysis and trial and error running based on the nature of the objective function and measured dataset.
**Algorithm 1.** Adapted SA method for data-driven system identification application1  Initialize the algorithm parameters: Cooling rate alfa, Temp. $T_{0}$, Boltzmann’s constant k 2  Initialize the input parameters ranges: Duffing coefficients’ variational range (m, alpha, delta, beta, gamma, omega) 3  **while** termination criterion is not satisfied **do** (reach final iterations = 300) 4      **for** i:M iteration without temperature change (M = 30) 5          Active Random mechanism to select the set of corresponding Duffing coefficient. 6          Make a new solution e.g., for only two parameters: ${beta}_{0}+∆beta$ & ${gamma}_{0}+∆gamma\;and\;so\;on\;based\;on\;step\;5$
7             **if** f(${beta}_{0}+∆beta$, ${gamma}_{0}+∆gamma$) > f(${beta}_{0}$, ${gamma}_{0}$) then 8                 fbest = f(${beta}_{0}+∆beta$ & ${gamma}_{0}+∆gamma$); 9                 ${beta}_{0}={beta}_{0}+∆beta$ & ${gamma}_{0}={gamma}_{0}+∆gamma$
10               **else** 11               random r(0, 1) (select random number) 12                   **if** r > exp(−∆f/kT) then (check the Boltzmann’s probability) 13                           f_best_ = f(${beta}_{0}+∆beta$ & ${gamma}_{0}+∆gamma$); 14                           ${beta}_{0}={beta}_{0}+∆beta$ & ${gamma}_{0}={gamma}_{0}+∆gamma$
15                           **else** 16                           f_best_ = f(${beta}_{0}$ & ${gamma}_{0}$), 17                   **End if** 18           **End if** 19    **if** abs((∆f (i −)) >= (abs(∆f (i −2))) && i > 5 20            sgn= −1*sgn; (change the movement direction) 21    **End if** 22    **End for** 23  T = alfa $\times T_{0}$ (applying a cooling procedure) 24  f = f_best_ 25 **End while**

### 2.3. Numerical Simulation

#### 2.3.1. Governing Equation

In this section, an overview of the governing equations utilized in the numerical model is presented. The model consists of two main components: the magnetic field generator and the microresonator. The magnetic field is modeled by a 2D multilayer spiral coil in the shell interface. Solving the electric currents in the shell problem enables the determination of the electric potential drop along the conductor. Subsequently, the lumped resistance (R) is computed using Ohm’s law.

(4) $$R=\frac{\cup }{I}$$

where, U represents the electric potential at the specific terminal, and I is the current flowing through it. The surface current density is then calculated and used as a source term in Ampère’s law to determine the magnetic field in the space surrounding the coil. By considering the total magnetic energy ($w_{m}$), the inductance can be formulated as follows:

(5) $$L=\frac{2w_{m}}{I^{2}}$$

The magnetic behavior of materials is simulated by utilizing Equation (6), which employs non-linear magnetostrictive models, relying on the homogeneity assumption of magnetic domain anisotropy energy [50].

(6) $$\lambda_{\theta }=\frac{3}{2}\lambda_{s}(cos(\theta {)}^{2}-\frac{1}{3})=\frac{3}{2}\lambda_{s}(\frac{M^{2}}{M_{s}^{2}}-\frac{1}{3})=\frac{3}{2}\frac{\lambda_{s}}{M_{s}^{2}}dev(M⊗M-\frac{1}{3})$$

where M is the dipole moment, $M_{s}$ is the saturation magnetization, $\lambda_{\theta }$ is the magnetostriction coefficient, $\lambda_{s}$ is the saturation magnetostriction, and cosine represents the trigonometric ratio between the dipole moment and saturation magnetization. Magnetostriction strain is determined under the assumption of a constant volume, considering solely the deviatoric component of the stress tensor. Modeling the nonlinear magnetization was achieved using the Langevin function and the following equations [50]:

(7) $$M=M_{s}L(\left|H_{eff}\right|)\frac{H_{eff}}{\left|H_{eff}\right|}$$

(8) $$H_{eff}=H+\frac{3\lambda_{s}}{\mu_{0}M_{s}^{2}}S_{ed}M$$

(9) $$S_{ed}=dev(C_{H}\epsilon_{el})$$

(10) $$L=coth(\frac{3\chi_{m}\left|H_{eff}\right|}{M_{s}})-\frac{M_{s}}{3\chi_{m}\left|H_{eff}\right|}$$

Here, *L* is the Langevin function, $\chi_{m}$ is the mass magnetic susceptibility, $S_{ed}$ is the deviatoric elastic tensor, and $C_{H}$ and $\epsilon_{el}$ are the elastic modulus tensor and elastic strain, respectively. Meanwhile, the magnetic field and magnetic flux can be calculated from the material susceptibility properties.

#### 2.3.2. Modeling Procedure

In this study, numerical modeling was conducted using the finite element method in COMSOL 6.1 software. The ambient conditions were simulated with an air domain element represented by a cubic shape. To avoid unwanted wall effects, an infinite element was applied to the external boundary of the air domain. The magnetic field was generated by a 2D multilayer spiral coil, coupling the electric currents in Shells physics interface with the Magnetic Field physics interface. The PCB coil was modeled using copper as the layered material with a thickness of 0.1 mm, while the PCB substrate utilized epoxy resin as an insulating layer between conducting layers. For the purposes of the model, the focus is on current conduction, and therefore, zero conductivity was applied to both the air material and the substrate. The excitation AC signal was applied to the coil via harmonic perturbation functions. The microresonator model comprises a stack of layers: a 2-μm AIN piezoelectric layer on top, a 50-μm Si polysilicon layer in the middle, and a 2-μm FeCoSiB magnetostrictive layer below. The length and width dimensions are set to 1 × 3 mm, which was aligned with the fabricated version. The material specifications used in the simulation can be found in Appendix A.

The discretization and meshing were implemented using custom size mapped and tetrahedral elements. The solving procedure involved coupling different physics, such as magnetic field and solid mechanics, to simulate the magnetostrictive effect and stress–strain interaction, respectively. The eigenvalue, frequency domain, and time domain solver were employed to assess the mode shapes, frequency responses, and dynamic behaviors of the microresonator.

### 2.4. Measurement Scheme

The displacement of the microresonator was measured using a laser Doppler vibrometer with sub-picometer detection accuracy. The laser spot was precisely aligned perpendicular to the microresonator surface, and 55 network points were selected for discrete measurement. Velocity was directly measured from the laser sensor, and integration was applied to calculate the displacement from the time domain signal. To enhance measurement accuracy and reduce the level of noise, each point was measured 10 times, and the mean value was considered. A 200 ms delay was implemented between each measurement to ensure reaching a steady-state condition. The vibration data were recorded using a 1.25 MHz sampling rate for the first bending mode around 7.5 kHz. To minimize noise interference, a digital bandpass filter with a range of 6–40 kHz was applied to the laser signal. Subsequently, the measured data were analyzed using both Polytec and MATLAB software to reconstruct the mode shape.

**Figure 2:**
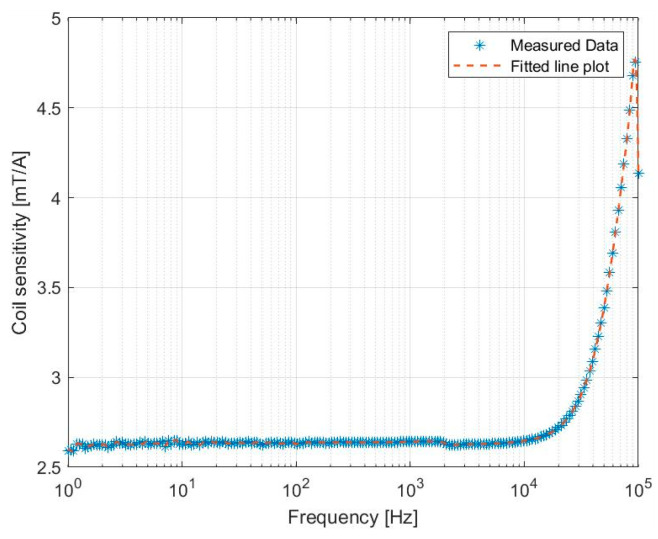
Experimental measurement of planar spiral coil sensitivity.

To convert the current to the magnetic field, the sensitivity of the spiral coil was measured within the kHz frequency range using a Tesla meter (FM302 AS-Lab). Figure 2 illustrates that within the 1–10 k frequency working range, the sensitivity remains nearly constant. Evidently, at 7.5 kHz, the sensitivity is approximately 2.6 mT/A.

**Figure 3:**
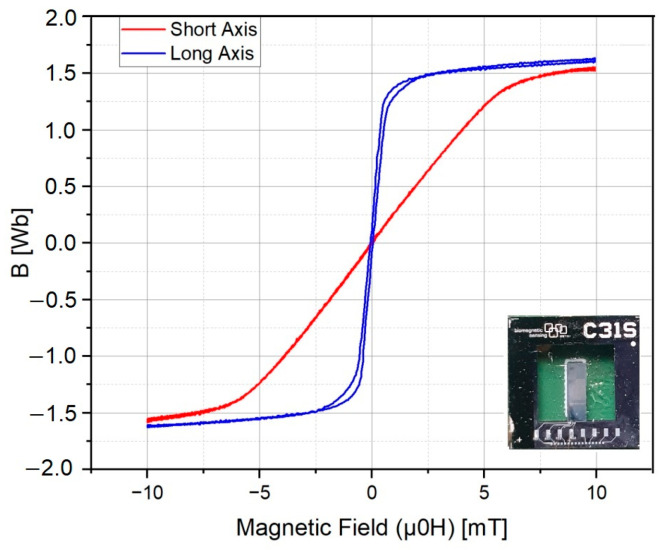
Magnetization measurement of the microresonator for both the short and long axis; inset illustrates the fabricated microresonator.

To model the magnetic behavior of the thin-film microresonator and assess the linear regime, magnetization was measured using a vibrating-sample magnetometer (VSM) device. The measurements were conducted over a range of 0–10 mT. As shown in Figure 3, the magnetic layer exhibits nonlinearity along the sensitive axis, which initiates at approximately 1 mT.

## 3. Results and Discussion

### 3.1. Numerical Model

**Figure 4:**
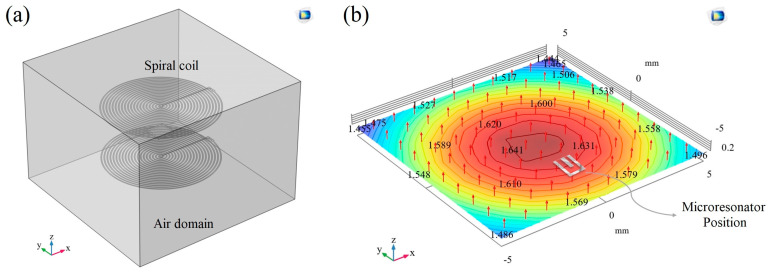
Simulation result; (**a**) embedded spiral coil in cubic air domain, (**b**) magnetic flux density contour (mT).

The excitation magnetic field serves as a driving force for the ME microresonator, significantly influencing its oscillation performance. To assess the degree of magnetic uniformity across the ME microresonator position, the magnetic flux density magnitude within the spiral coil area has been calculated using a numerical simulation method in the cubic air domain (Figure 4a). As shown in Figure 4b, a 98% homogeneity is attainable along the longitudinal axis.

**Figure 5:**
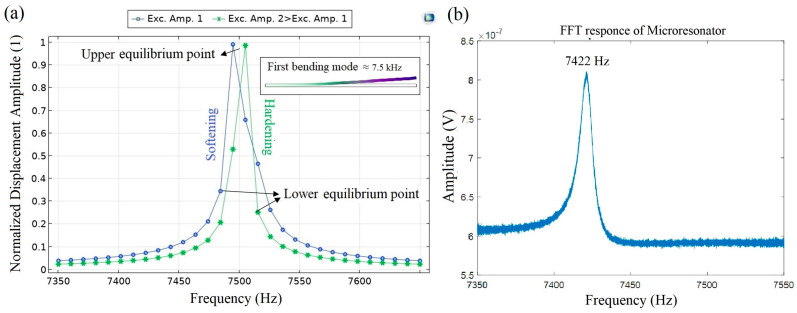
Frequency response of microresonator; (**a**) simulation results under different excitation fields with an inset illustrating the eigenfrequency response for the first bending mode, (**b**) experimental result of the output signal for the first bending mode.

The magnetoelectric microresonator could manifest nonlinear behaviors when the excitation amplitude exceeds a certain threshold. In such scenarios, the oscillator undergoes detuning, causing the frequency response to shift asymmetrically. The threshold for reaching nonlinearity was around 1 mT based on the experimental results (Figure 3). Figure 5a illustrates the frequency response of the microresonator for different magnetic excitation amplitudes, selected based on experimental measurements. The inset within Figure 5a presents the outcome of the clamp-free eigenfrequency analysis. The resonance frequency occurred around 7.5 kHz, aligning reasonably with the experimental results (Figure 5b). As can be seen, when the excitation amplitude remains below a certain value, the resonance shifts to lower frequencies, and after that it increases to higher frequencies. This transition in resonance frequency can be attributed to the increasing influence of nonlinearity in the oscillator’s behavior. Specifically, as the symmetric frequency response experiences a pronounced tilt towards lower frequencies, the heightened nonlinear contribution results in a softening of the resonator, leading to a reduction in the resonance frequency. Conversely, when the frequency response tilts towards higher frequencies, the resonator exhibits a hardening behavior, indicating an elevation in the resonance frequency. Notably, points of discontinuity within the frequency behavior indicate an unstable regime for the resonator, denoted by the accompanying arrows. This frequency-shifting phenomenon observed in magnetoelectric oscillators is in line with earlier investigation [33].

**Figure 6:**
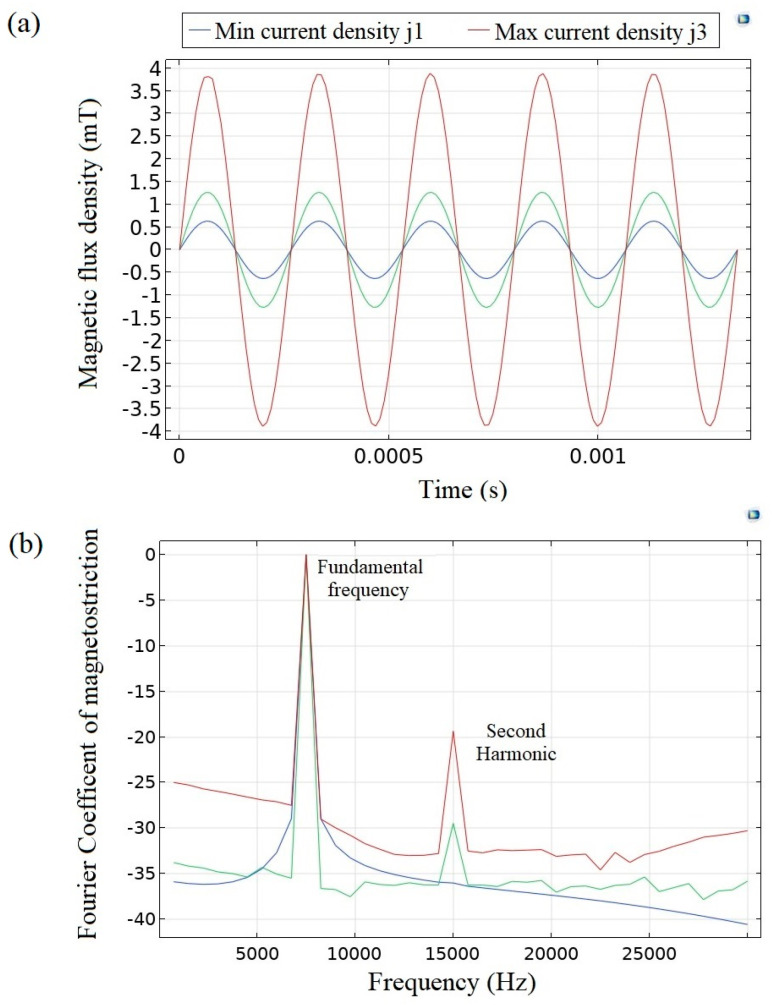
Simulation results for microresonator magnetostriction under different excitation fields; (**a**) amplitude of applied magnetic field at the oscillator’s tip point, (**b**) fast Fourier transform analysis of time domain magnetostriction signal.

Developing a numerical model to predict the nonlinearity behavior of the thin-film oscillator is a formidable task due to the computational expense incurred by the mesh aspect ratio issue. In this context, the model has been solved for only five oscillation periods using a time domain solver. Figure 6a displays the magnetic field amplitude at the microresonator’s evaluation point, ranging from 0.5 to 3.5 mT. The assessment of magnetostriction for the first bending mode was calculated on the Z component of the magnetostrictive tensor. Subsequently, Figure 6b illustrates the fast Fourier transform (FFT) of the simulated magnetostriction signal. Remarkably, the onset of nonlinearity becomes evident at a threshold, roughly around 1 mT, validating the model’s performance. The total harmonic distortion (THD) increases as the driving force grows. In the simulation, the signal is originally pure and monofrequency so the observed nonlinearity stems from the properties of the magnetostrictive layer and material characterization.

### 3.2. Data-Driven Model

**Figure 7:**
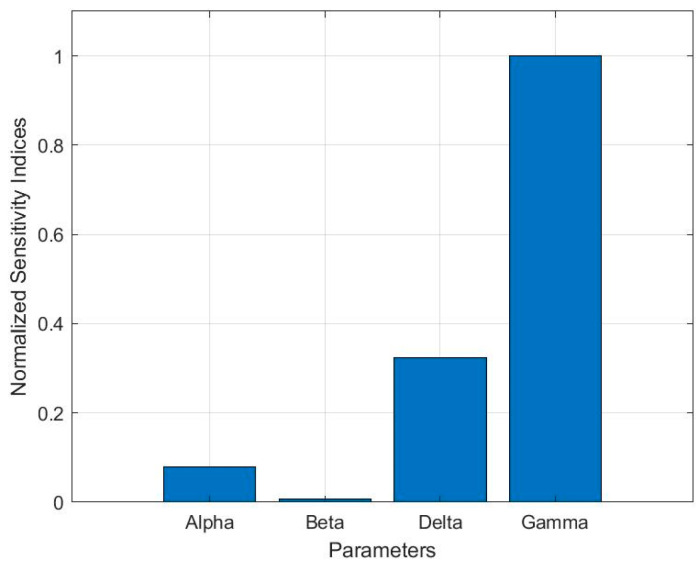
Normalized sensitivity analysis for Duffing equation.

The focus of this section is to reconstruct governing equations from the time series dataset obtained during the excitation of the ME microresonator. The experimental setup (Figure 1) has been utilized to measure both vibration and velocity signals. The simulated annealing algorithm (SA) has been employed to reconstruct the governing equations of the experimental system. The first assessment focused on measuring the displacement at the cantilever tips. With the aim of enhancing processing speed, data reduction has been applied to the measured dataset, resulting in a reduction in the sampling rate to 125 kHz. The recorded signal, lasting for 1 s, underwent reconstruction over 187 s, using the SA algorithm with 300 iterations over an i7-intel CPU. The objective function and the current optimization problem are well-posed, implying the existence of a unique solution. Nevertheless, the problem may exhibit ill-conditioning, implying that even a slight perturbation in the system’s input could lead to significantly magnified errors in the resulting solutions. To address this, a global sensitivity analysis was performed to ascertain the appropriate range and step size for each Duffing coefficient within the SA algorithm’s framework. This analysis aids in understanding how perturbations in these coefficients impact the system’s behavior and facilitates the selection of optimal parameter intervals and incremental values for the SA optimization process. Consequently, the Duffing equation has been solved for various input parameter combinations using the Latin hypercube sampling (LHS) method. The sensitivity indices were then determined using the computed normalized output values through the finite differences method (FDM), with a minor perturbation size of approximately 1 × 10^−5^. As depicted in Figure 7, the driving force amplitude, identified as Gamma, and the stiffness parameter, termed Delta, exhibit the highest levels of sensitivity, which has been considered in the determination of the step size.

The applied driving force frequency is known. However, from a practical point of view, there exists a slight discrepancy between the desired frequency and the measured applied frequency. This discrepancy has been incorporated into the SA algorithm as an input parameter, Omega, constrained within a limited range of variation. The initial temperature of the SA algorithm has been determined through 5 executions of the algorithm, using the average value of the delta function. This parameter prioritizes the acceptance of solutions without improvement during the initial stages. Afterward, the cooling factor has been adjusted to progressively lower the acceptance rate every 30 iterations, leading to a controlled reduction in the acceptance rate value. To ensure numerical accuracy, different objective functions including peak–peak amplitude (PP) of error, peak index matching (PIM), and correlation coefficient (CC) were considered as the figure of merit in the SA algorithm. 

**Figure 8:**
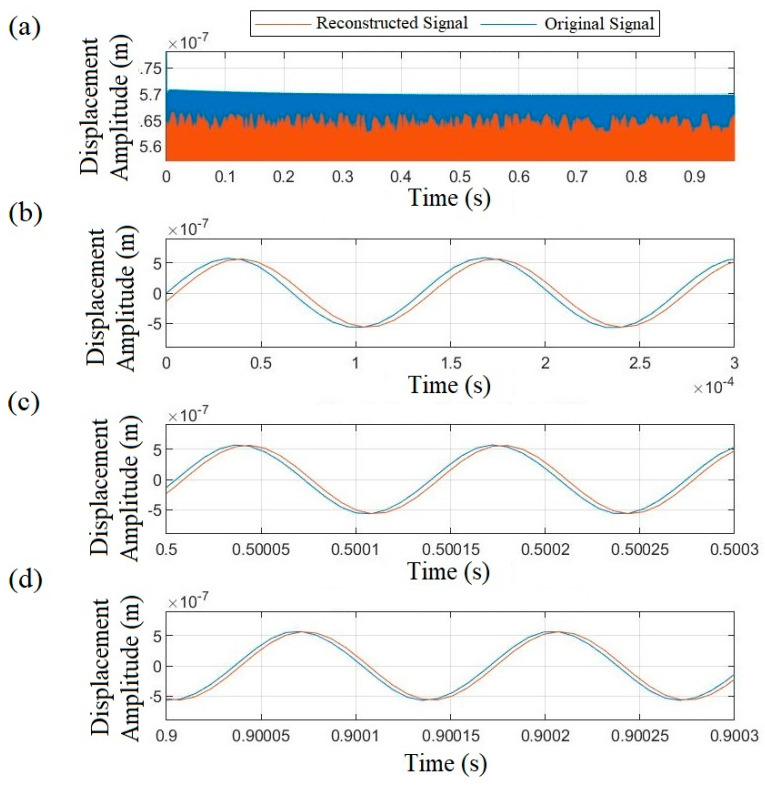
(**a**) **Amplitude** Comparison between measured displacement and reconstructed Duffing equation solutions across multiple time intervals including (**b**) first, (**c**) mid, and (**d**) last slot of time domain (driving force 0.28 mT).

The reconstructed Duffing equation has been solved over the time domain with a 125 kHz sampling rate in line with the recorded signal. Figure 8 depicts a comparison between the original and reconstructed signals across different time slots. As can be seen, for the entire signal duration a minor discrepancy in amplitude matching is observed in the range of 4 × 10^−9^ or less than a 2 percent error (Figure 8a). Additionally, Figure 8b–d demonstrate strong consistency in the signal phase. The high correlation coefficient of 0.995 between the two signals confirms the accuracy of the model. It should be noted that the governing model was specifically developed based on recorded data from the fabricated exchange ME microresonator. However, the same measurement procedure and proposed algorithm could potentially be applied for modeling other types of ME resonators.

**Figure 9:**
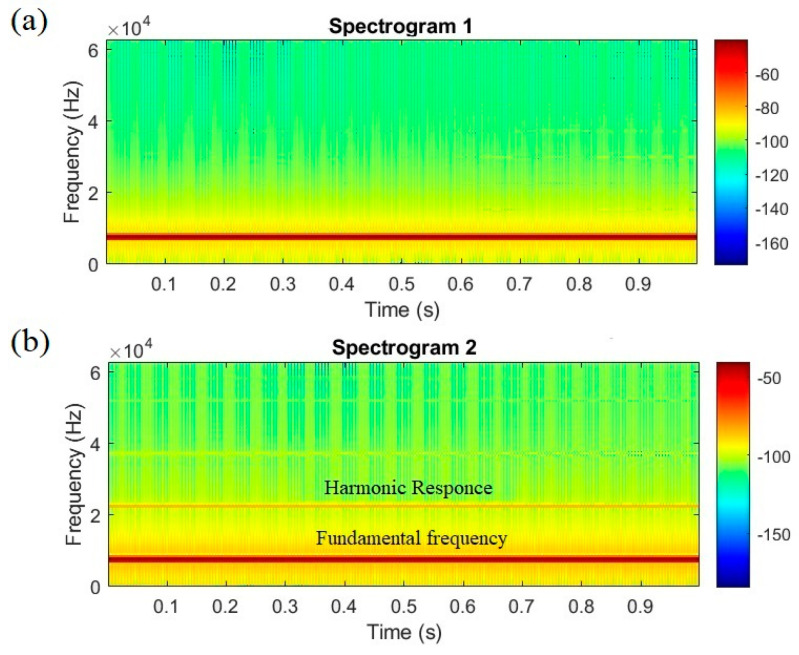
Solution of the reconstructed equation with (**a**) 0.28 mT and (**b**) 0.8 mT driving force amplitudes.

Displacement measurements have been taken across various amplitudes, subsequently allowing for the reconstruction of the Duffing equation. The results reveal that under higher driving forces, the “beta” term in the Duffing equation becomes evident in the reconstructed model, leading to distortions in the time domain signal. Figure 9 illustrates the comparison between the short-time Fourier transform of the reconstructed signals for two distinct amplitudes. As shown in Figure 9a, the entire signal exhibits a monofrequency, indicating a clear linear response of the microresonator. In Figure 9b, the initial signs of nonlinearity occur in the reconstructed signal, proved by the presence of a weak harmonic response.

**Figure 10:**
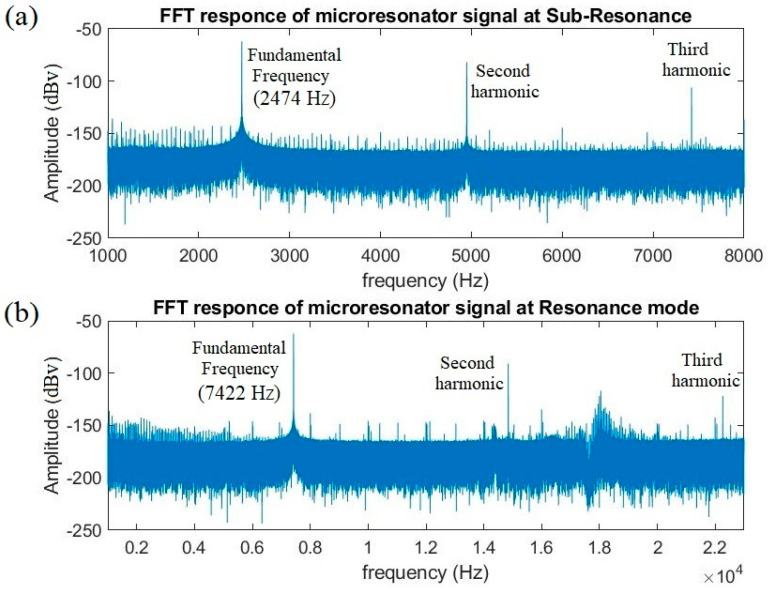
The microresonator response under (**a**) sub-resonance and (**b**) resonance mode excitation signal.

To effectively design an ME microresonator, it is essential to consider various contributors to nonlinearity. These sources encompass both the nonlinearity of the excitation signal and the material itself. When employing an AC excitation scheme, particularly at high amplitudes and high frequencies, generating a pure magnetic field proves challenging, due to hardware limitations. In this context, the linearity of the excitation signal has been initially quantified by direct measurement before its application to the microresonator. Subsequently, the output voltage of the ME resonator was measured to assess its response. The FFT analysis has been performed on the recorded signals using the Han filter at the 469 kHz sampling rate. Every measurement has been repeated 30 times to improve accuracy, with the average value reported. Figure 10a,b provide a visual representation of the microresonator’s response under distinct conditions: resonance excitation and sub-resonance excitation, both at a magnetic field strength of 0.28 mT. As can be seen, even with low excitation levels, the signal displays discernible higher harmonic components. The THD values for resonance and sub-resonance excitation are 2.16 and 2.48, respectively. This observation emphasizes the significance of harmonic distortion and source nonlinearity, especially in sub-resonance mode. This type of source noise could potentially be harnessed as a dual- or triple-oscillation source, enhancing the actuator’s bandwidth within a nonlinearity regime and being considered as an advantage [22],[23]. However, for applications such as weak signal detection, this mentioned nonlinearity could be challenging to deal with.

**Figure 11:**
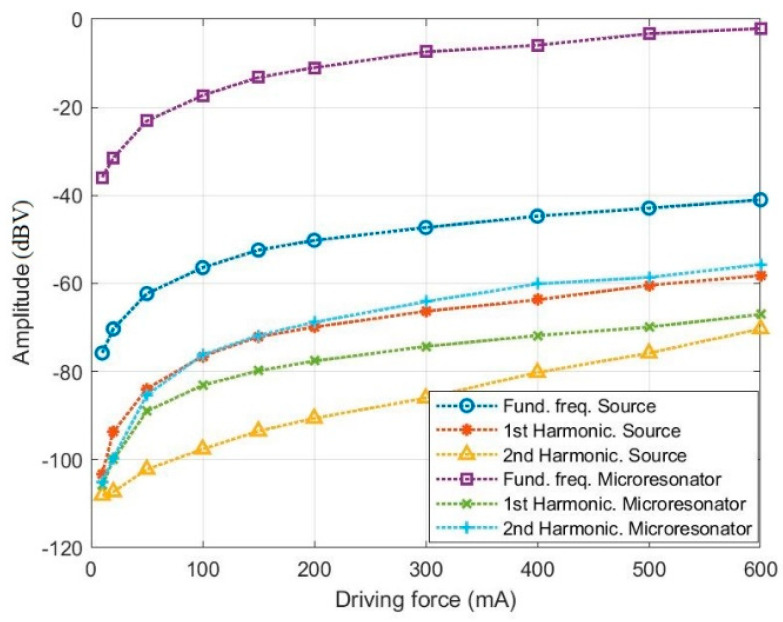
The harmonic response measurement at resonance mode, about 7.5 kHz.

Figure 11 illustrates the harmonic response of both the source signal and microresonator voltage at resonance mode. As illustrated, the mechanical quality factor of the microresonator amplifies the fundamental components of the frequency by a factor of about 6.5. This behavior is crucial to consider in weak signal detection due to the escalating impact of the nonlinearity issue in sub-harmonic excitation. Evidently, the level of nonlinearity in the source signal progressively intensifies, reaching a critical point for higher amplitudes of the driving force. This phenomenon directly affects actuator/sensor performance and should be a key consideration in the design process. It should be noted that at the high magnetic fields, the resonance frequency of the ME resonator will shift, due to the nonlinear magnetization. However, here the comparison was conducted at a fixed frequency to ensure a more consistent basis for evaluating varying amplitudes, spanning from the linear to nonlinear regimes.

**Figure 12:**
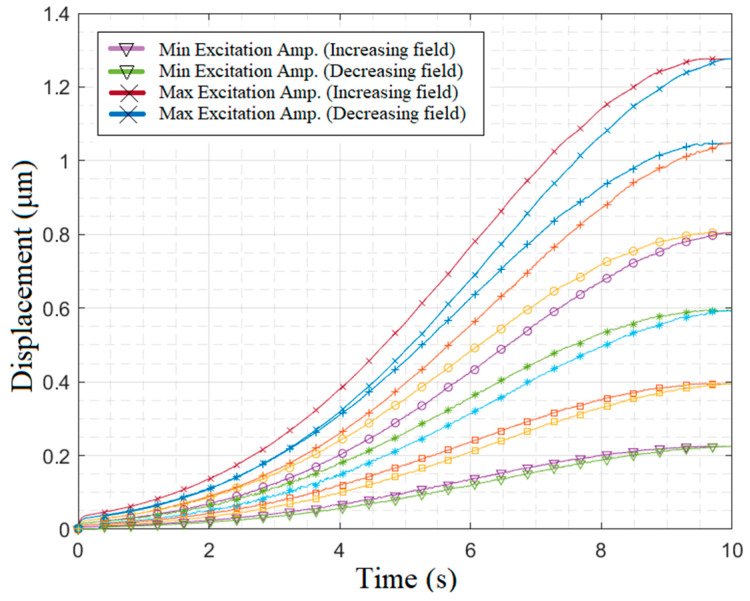
Investigation of hysteresis effects on microresonator displacement in resonance mode through experimental measurements.

The vibration amplitude of the ME microresonator has been assessed across varying driving force amplitudes. To achieve this, diverse magnetic field strengths were applied to the microresonator using a half-Gaussian function for a 10-s duration at the resonance frequency. As depicted in Figure 12, a noticeable hysteresis effect emerges at higher driving force levels. It is important to highlight that the hysteresis, attributed to the remanent magnetic field and magnetic wall domains in the magnetostrictive layer, cannot be modeled by the reconstructed Duffing equation. However, for lower excitation fields, the hysteresis effect is negligible and exerts minimal influence on prediction accuracy.

**Figure 13:**
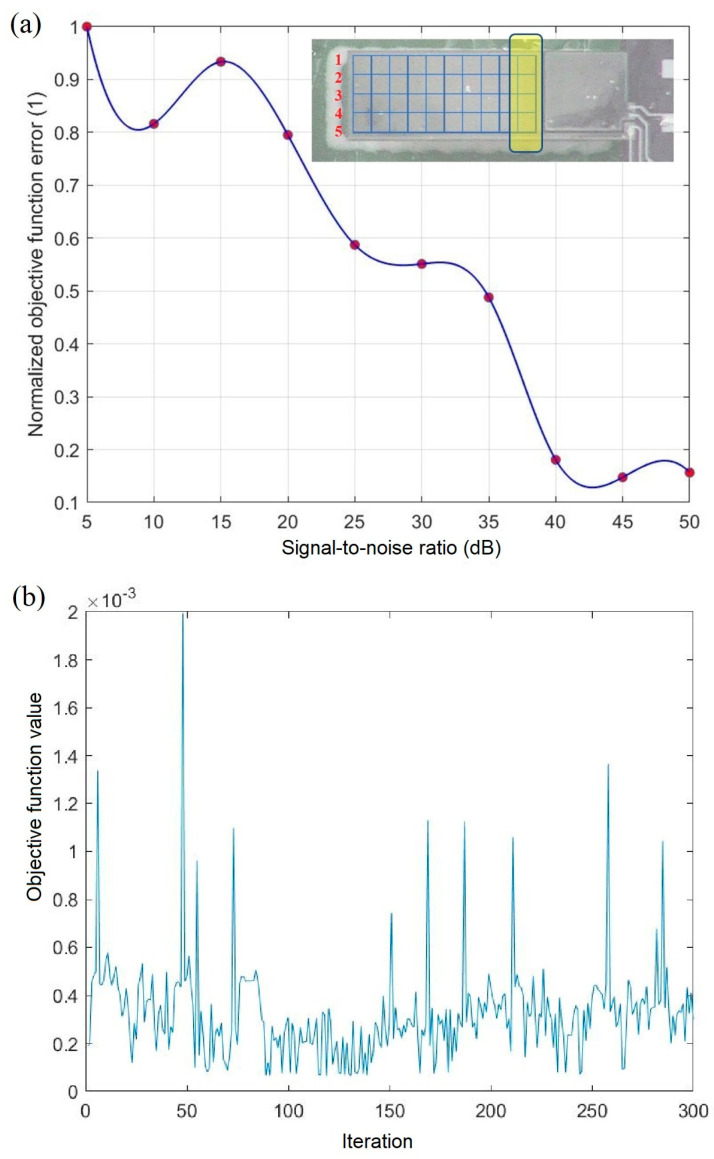
Numerical evaluation of SA algorithm performance, (**a**) effect of S/N ratio on error function, (**b**) convergence behavior of SA algorithm during data reconstruction at low S/N ratio.

Accurate mode shape reconstruction of microactuators plays a pivotal role in precise actuator design. Employing a Duffing equations array enables effective control strategies, and enhances system reliability, which is particularly crucial in micro-positioning applications [8,9]. In order to predict the vibration amplitude across the cantilever surface over the time domain, the 55 points on the microresonator surface have been selected and measured separately in a steady-state situation. In the initial step, the impact of noise level on the SA algorithm’s performance has been numerically evaluated for each point. As depicted in Figure 13a, achieving optimization below a 30 dB S/N level poses a challenge. Figure 13b shows that for low SNR values, due to the ill-conditioned nature of the Duffing oscillator, the algorithm fails to converge even for 300 iterations. The driving force amplitude has been experimentally chosen to achieve robust signal-to-noise levels for all points, including those in proximity to the clamp area, as highlighted in the inset of Figure 13a.

**Figure 14:**
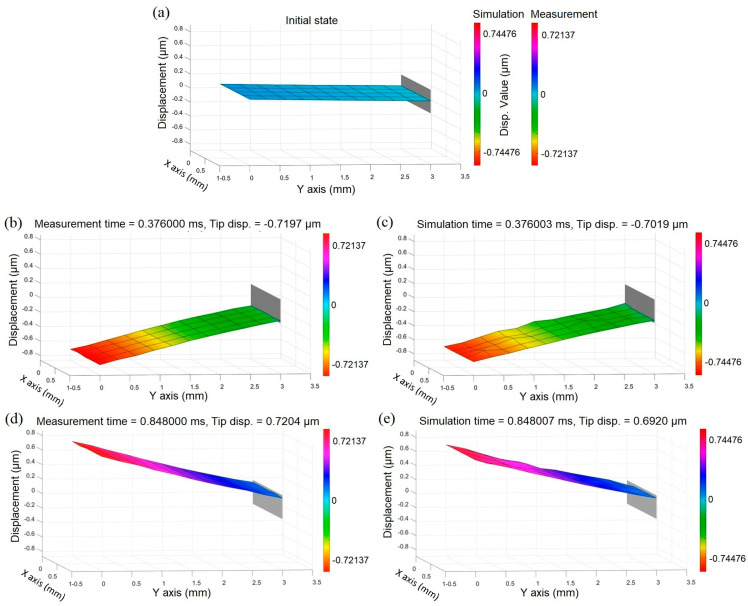
Mode shape reconstruction; (**a**) initial state of the first bending mode, (**b**,**c**) measured mode shape using laser Doppler vibrometer, (**d**,**e**) reconstructed mode shape using Duffing oscillator array.

The Duffing oscillator array has been trained using a driving force given by a magnetic field of 0.28 mT and subsequently solved for 0.4 mT to reconstruct the first bending mode shape. For more details regarding the reconstructed coefficients at different points, please refer to Appendix B. To validate the simulation results, microresonator vibrations were recorded using an LDV device at the resonance frequency. The mode shape reconstruction results are illustrated in Figure 14a–e. Moreover, the video of the comparison between measurement and simulation results can be found in the Supplementary Materials (Video S1). In Figure 14a, it is clear that the simulated vibration range spans ±0.74476 µm, exceeding the actual measurement values by 3 percent. The accuracy of the developed Duffing equation varied for each point based on the SNR level, yielding an average correlation coefficient (CC) of around 0.92 percent. Although the vibration amplitude within the upper and lower ranges closely matched experimental data, certain border points exhibited lag responses, as shown in Figure 14c,e, causing surface distortion. Reconstructing intricate mode shapes using the Duffing array is achievable, subject to practical constraints such as the number of network points, SNR value, and LDV dimensional measurements along different axes. To encompass the spectrum from linear to nonlinear behavior, training the Duffing array with a step Gaussian function is recommended and will be explored in upcoming research. Emphasizing the Duffing oscillator model’s limitation, it is incapable of predicting hysteresis effects. Nevertheless, for minimal hysteresis, the developed model is useful in closed-loop feedback systems, enabling time-domain control of the MEMS system.

## 4. Conclusions

This study introduces a new approach to modeling magnetoelectric microresonators through data-driven system identification and a simulated annealing algorithm. It investigates the dynamic behavior of the Cr–FeCoSiB/AIN thin-film ME microactuator, particularly in its first bending mode, exploring both linear and nonlinear domains. The displacement vibration is described by a Duffing equation within a single degree-of-freedom (DOF) system. A magnetic driving force is generated by a spiral coil, ensuring 98 percent magnetic homogeneity. Coil sensitivity measurements reveal consistent sensitivity within the operational range, with a transfer factor of approximately 2.6 mT/A. The microresonator’s displacement was precisely measured using a laser Doppler vibrometer device at 7.5 kHz. Nonlinearity’s impact on the microresonator’s response was explored through finite element (FE) simulations, revealing significant nonlinearity emerging after reaching 1 mT, in alignment with VSM magnetization measurements. To reconstruct dynamic behavior using the Duffing oscillator, input parameter ranges for the simulated annealing optimization algorithm were established through global sensitivity analysis. Comparing measured displacement with reconstructed Duffing equation solutions indicated a model precision of approximately 98 percent. The study highlights challenges faced by the simulated annealing algorithm when converging for boundary points on the cantilever with signal-to-noise ratios (SNR) below 30 dB. an examination of the microresonator’s response underscores the importance of harmonic distortion and source nonlinearity, particularly in the sub-resonance mode, due to the quality factor of the ME resonator. Vibration measurements revealed significant hysteresis variations at higher excitation levels, which impact the prediction precision of the developed Duffing array. Nonetheless, minimal hysteresis values at lower excitation levels enable reasonable reconstruction and prediction of the first bending mode shape, with an average correlation coefficient of approximately 92 percent. In conclusion, the proposed method is a promising tool for dynamic behavior analysis of MEMS actuators. It effectively contributes to predicting mode shapes and displacement profiles over time, which significantly enhances the precision of MEMS actuator design and control. Further research is required to explore the contributions of hysteresis effects, the sensitivity axis angle, temperature, and complex mode shape reconstruction.

## Data Availability

The data that support the findings of this study are available from the corresponding author upon request.

## Supplementary Materials

The following supporting information can be downloaded at: https://www.mdpi.com/article/10.3390/mi14101878/s1, Video S1: Comparison between measured and predicted mode shape in the time domain.

## Abbreviations

MEMS	Micro-electromechanical systems
ME	Magnetoelectric
SA	Simulated Annealing
LDV	Laser Doppler Vibrometer
THD	Total Harmonic Distortion
STFT	Short-time Fourier Transform
FE	Finite Element
SINDy	Sparse Identification of Nonlinear Dynamical Systems
ANN	Artificial Neural Network
DOF	Degree-of-Freedom
VSM	Vibrating-Sample Magnetometer
LHS	Latin hypercube sampling
FDM	finite differences method
RMS	Root mean square
PIM	Peak Index Matching
CC	Correlation Coefficient
z	Out-of-plane displacement
ζ	Damping ratio
α	Linear stiffness
γ	Non-linear stiffness
μ	Non-linear damping
$F_{ri}$	Amplitude of the desired stimulation force
ω	Angular frequency of the desired stimulation force
f(x)	Objective function
T	Temperature
k	Boltzmann’s constant
∆f	Difference between objective function for every iteration
U	Electric potential
I	Current
*L*	Surface current density
$w_{m}$	Total magnetic energy
M	Dipole moment
$M_{s}$	Saturation magnetization
$\lambda_{\theta }$	Magnetostriction coefficient
$\lambda_{s}$	Saturation magnetostriction
L	Langevin function
$\chi_{m}$	mass magnetic susceptibility
$S_{ed}$	Deviatoric elastic tensor
$C_{H}$	Elastic modulus tensor
$\epsilon_{el}$	Elastic strain

## Appendix A

Material parameters [51,52]:

$$\rho_{silicon}=2329\;[\frac{kg}{m^{3}}]$$

$$\upsilon_{silicon}=0.28\;[1]$$

$$\sigma_{silicon}=1^{-12}\;\left[S/m\right]$$

$$C_{silicon}^{EH}=\left[\begin{array}{cccccc} 216 & 84 & 84 & 0 & 0 & 0\\ 84 & 216 & 84 & 0 & 0 & 0\\ 84 & 84 & 216 & 0 & 0 & 0\\ 0 & 0 & 0 & 66 & 0 & 0\\ 0 & 0 & 0 & 0 & 66 & 0\\ 0 & 0 & 0 & 0 & 0 & 66 \end{array}\right]\;Gpa$$

$$\mu_{Air}=1$$

$$\epsilon_{Air}=1$$

$$\sigma_{Air\;\&\;AIN}=1^{-12}\;[S/m]$$

$$\rho_{AIN}=3268\;[\frac{kg}{m^{3}}]$$

$$C_{AIN}^{EH}=\left[\begin{array}{cccccc} 410 & 149 & 99 & 0 & 0 & 0\\ 149 & 410 & 99 & 0 & 0 & 0\\ 99 & 99 & 389 & 0 & 0 & 0\\ 0 & 0 & 0 & 125 & 0 & 0\\ 0 & 0 & 0 & 0 & 125 & 0\\ 0 & 0 & 0 & 0 & 0 & 125 \end{array}\right]\;Gpa$$

$$\upsilon_{AIN}=0.3\;[1]$$

$$\varepsilon_{AIN}=\left[\begin{array}{ccc} 80 & 0 & 0\\ 0 & 80 & 0\\ 0 & 0 & 80 \end{array}\right]\;pF/m$$

$$\sigma_{FeCoSiB}=0.833333\;[MS/m]$$

$$\rho_{AIN}=7250\;[\frac{kg}{m^{3}}]$$

$$M_{s\;FeCoSiB}=159.15\;[KA/m]$$

$$\chi_{FeCoSiB}=40$$

$$\upsilon_{FeCoSiB}=0.33\;[1]$$

$$C_{FeCoSiB}^{EH}=\left[\begin{array}{cccccc} 150 & 45 & 45 & 0 & 0 & 0\\ 45 & 150 & 45 & 0 & 0 & 0\\ 45 & 45 & 150 & 0 & 0 & 0\\ 0 & 0 & 0 & 40 & 0 & 0\\ 0 & 0 & 0 & 0 & 40 & 0\\ 0 & 0 & 0 & 0 & 0 & 40 \end{array}\right]\;Gpa$$

## Appendix B

**Table A1:** Reconstructed Duffing coefficients for 0.4 mT driving force.

	Parameter	Delta	Alpha	Omega	Gamma	Mass
Points Number						
1		0.361671999593	0.278040544940	4.66328953897 × 10^4^	0.339451442238	0.065870878718
2		0.363013075806	0.309101936376	4.66334492152 × 10^4^	0.337459733144	0.064703821094
3		0.362175254191	0.302470915090	4.66330440760 × 10^4^	0.337614422055	0.065506621181
4		0.362394000000	0.303695603594	4.66330521002 × 10^4^	0.34024500000	0.06593900000
5		0.363506320901	0.283383704287	4.66333831975 × 10^4^	0.332648966731	0.064578975393
6		0.362433330694	0.299659422394	4.66330872224 × 10^4^	0.340984715495	0.06593900000
⋮		⋮	⋮	⋮	⋮	⋮
52		0.361974686843	0.330977245251	4.66330944730 × 10^4^	0.336451973259	0.066092226877
53		0.362475601632	0.296297000000	4.6633521067 × 10^4^	0.3402450000	0.0661028238
54		0.3621115925123	0.299697723699	4.66331662856 × 10^4^	0.3387779527	0.0647517934
55		0.362820938412	0.360317841738	4.6633158936 × 10^4^	0.3553373737	0.0683564117

Beta was detected as zero for all points based on the force amplitude. All output values have been scaled for SA convergence improvement.
